# The calcimimetic R-568 induces apoptotic cell death in prostate cancer cells

**DOI:** 10.1186/1756-9966-28-100

**Published:** 2009-07-14

**Authors:** Huaifu Li, Guofeng Ruan, Zhijun Li, Ziwei Liu, Xiaoqing Zheng, Hao zheng, Guangming Cheng, Benyi Li, Ming Zhan

**Affiliations:** 1 Department of Urology, The Fifth Affiliated Hospital of Sun Yat-sen University, Zhuhai 519000, PR China; 2 Department of Gynecology and Obstetrics, The Fifth Affiliated Hospital of Sun Yat-sen University, Zhuhai 519000, PR China; 3 Department of Urology, The University of Kansas Medical Center, Kansas City, KS 66160, USA

## Abstract

**Background:**

Increased serum level of parathyroid hormone (PTH) was found in metastatic prostate cancers. Calcimimetic R-568 was reported to reduce PTH expression, to suppress cell proliferation and to induce apoptosis in parathyroid cells. In this study, we investigated the effect of R-568 on cellular survival of prostate cancer cells.

**Methods:**

Prostate cancer cell lines LNCaP and PC-3 were used in this study. Cellular survival was determined with MTT, trypan blue exclusion and fluorescent Live/Death assays. Western blot assay was utilized to assess apoptotic events induced by R-568 treatment. JC-1 staining was used to evaluate mitochondrial membrane potential.

**Results:**

In cultured prostate cancer LNCaP and PC-3 cells, R-568 treatment significantly reduced cellular survival in a dose- and time-dependent manner. R-568-induced cell death was an apoptotic event, as evidenced by caspase-3 processing and PARP cleavage, as well as JC-1 color change in mitochondria. Knocking down calcium sensing receptor (CaSR) significantly reduced R-568-induced cytotoxicity. Enforced expression of Bcl-xL gene abolished R-568-induced cell death, while loss of Bcl-xL expression led to increased cell death in R-568-treated LNCaP cells,.

**Conclusion:**

Taken together, our data demonstrated that calcimimetic R-568 triggers an intrinsic mitochondria-related apoptotic pathway, which is dependent on the CaSR and is modulated by Bcl-xL anti-apoptotic pathway.

## Introduction

Calcimimetic agents, like NPS R-568 (Cinacalcet HCl), is an allosteric agonist for parathyroid calcium-sensing receptor (CaSR) and was shown to lower circulating levels of parathyroid hormone (PTH) in patients with secondary hyperparathyroidism due to late-stage renal diseases [reviewed in [1,2]]. In addition, studies have shown that CaSR is involved in cell differentiation and apoptosis in osteoblast cells [3] and NPS R-568 treatment induced apoptotic cell death in hyperplastic parathyroid cells [4].

In the literature, clinical reports have shown that increased levels of serum PTH was frequently found in advanced prostate cancers [reviewed in ref. [5]], since the first description of possible secondary hyperparathyroidism (SHPT) as an accompanied syndrome with late-stage prostate cancer patients more than 46 years ago [6]. In theory, osteoblastic lesion in skeletal sites of metastatic prostate cancer causes hypocalcemia that in turn leads to calcium-sensing receptor (CaSR) activation, resulting in increased PTH production and secretion [5,6]. Meanwhile, PTH has been shown to increase cell proliferation of human prostate cancer *in vitro *[7] and to promote bone metastasis in mouse xenograft model of prostate cancer [8]. Therefore, reducing PTH secretion could potentially interrupt SHPT and be of substantial clinical benefit in prostate cancer patients.

In fact, a functional CaSR was detected in human prostate cancer cells [9,10]. However, the biological effect of calcimimetic agents on prostate cancer cells has not been evaluated. Therefore, in this study, we tested the biological effect of calcimimetic agent NPS R-568 on multiple prostate cancer cells. We surprisingly found for the first time that NPS R-568 induced apoptotic cell death, which is dependent on the CaSR and is modulated by anti-apoptotic Bcl-xL pathway.

## Materials and methods

### Cell Culture, Reagents and Antibodies

Human prostate cancer PC-3 and LNCaP, as well as LNCaP sublines (LNCaP/Bclxl and LNCaP/LN11) were described in our previous publication [11]. Briefly, LNCaP/Bclxl cells were established by stable transfection of LNCaP cells with a vector bearing HA-tagged human bcl-xl cDNA sequence (pcDNA3.1-Bclxl.HA). LN11 is a LNCaP cell subline that lost Bcl-xL expression, as described [11]. Cells were maintained in a humidified atmosphere of 5% CO_2_, RPMI 1640 supplemented with 10% fetal bovine serum (FBS) with antibiotics (Invitrogen, Carlsbad, CA). Antibodies for PARP, caspase-3, CaSR and Actin were purchased from Santa Cruz Biotech (Santa Cruz, CA). CaSR small interference RNA (siRNA) mixture and the negative control siRNA were obtained from Santa Cruz Biotech. The calcimimetic R isomer of N-[3-[2-chlorophenyl]propyl]-[R]-α-methyl-3-methoxybenzylamine (NPS R-568) and its inactive isomer NPS S-568 were kindly provided by Amgen, Inc. (Thousand Oaks, CA).

### Cell Viability Analyses

For MTT [3-[4,5-dimethylthazol-2-yl]-2,5-diphenyl tetrazolium-Bromide] assay, which is based on the conversion of MTT to MTT-formazan by mitochondrial enzyme, a cell growth determination kit (Sigma Co., St Louse, MO) was utilized according to the instruction from the manufacturer. Briefly, cells were seeded at a density of 2 × 10^3 ^cells/well in 96-well plates in triplicates and allowed to attachment overnight. Cells were then maintained in various conditions as indicated in the figures. The MTT solution was added in an amount equal to 10% of the culture volume. After 3 h incubation, the culture media was removed and the MTT solvent was added. The plates were read at a wavelength of 570 nM.

For trypan blue assay, cells were seeded in 12-well plates, and then treated with various reagents as indicated in the figures. At the end of experiments, viable cells was counted using a hemocytometer after staining with trypan blue as described in our recent publication [11].

For siRNA transfection, cells were plated in 6-well plates and transfected with the siRNA mixture as indicated in the figure using OligoTransfectamine™ (Invitrogen, Carlsbad, CA), as described in our previous publication [11]. Three days after transfection, cells were treated with the R568 at the concentrations indicated in the figure. Cellular survival was assessed with trypan blue exclusion assay.

To assess the cell death objectively, a LIVE/DEAD^® ^Viability/Cytotoxicity kit (Invitrogen, Carlsbad, CA) was utilized. This kit provides two molecular probes, of which one probe labels the living cells as green based on an intracellular esterase activity and the other probe simultaneously labels the dead cells as red due to the disruption of plasma membrane integrity. The assay was conducted by following the protocol provided by the manufacturer. Briefly, cells were placed in 24-well plates overnight, and treated with R-568 for different time periods as indicated in the figures. At each time points, cells were incubated with the fluorescent dyes (2.0 μM) for 15 min before micro-images were taken under a fluorescent microscope.

### Mitochondrial Membrane Potential (JC-1) assay

To examine the change of mitochondria membrane potential, JC-1 staining assay was used, as described in our previous publication [11]. Briefly, after treatment with R-568 or S-568 for 24 h, cells were incubated in the presence of JC-1 (Cell Technology Inc., Mountain View, CA) at a final concentration of 0.3 μg/ml for 15 minutes at 37C. Thereafter, the cells were analyzed under a fluorescent microscope.

### Western Blot Analysis

Western blot was carried out as described previously [11]. Briefly, cells were pelletted and lysed in a buffer containing protease inhibitors (Half™ Protease Inhibitor Cocktail Kit, PIERCE, Rockford, IL). Equal amounts of proteins were separated on SDS-PAGE gels and transferred to PVDF membrane (BIO-RAD, Hercules, CA). Membranes were blocked in a Tris-buffered solution plus 0.1% Tween 20 (TBS-T) solution with 5% nonfat dry milk and incubated with primary antibodies overnight at 4C. Immunoreactive signals were detected by horseradish peroxidase-conjugated secondary antibodies and chemiluminescence substrate purchased from (Santa Cruz Biotech., Santa Cruz, CA).

### Statistical Analysis

All cell culture-based experiments were repeated two or three times. Western blots are presented from representative experiments. The mean and SEM for cell viability assay are shown. The significant differences between groups were analyzed as described in our previous publication [11], using the SPSS computer software (SPSS Inc., Chicago, IL).

## Results

### The calcimimetic R-568 but not S-568 induces cell death in prostate cancer cells

**Figure 1:**
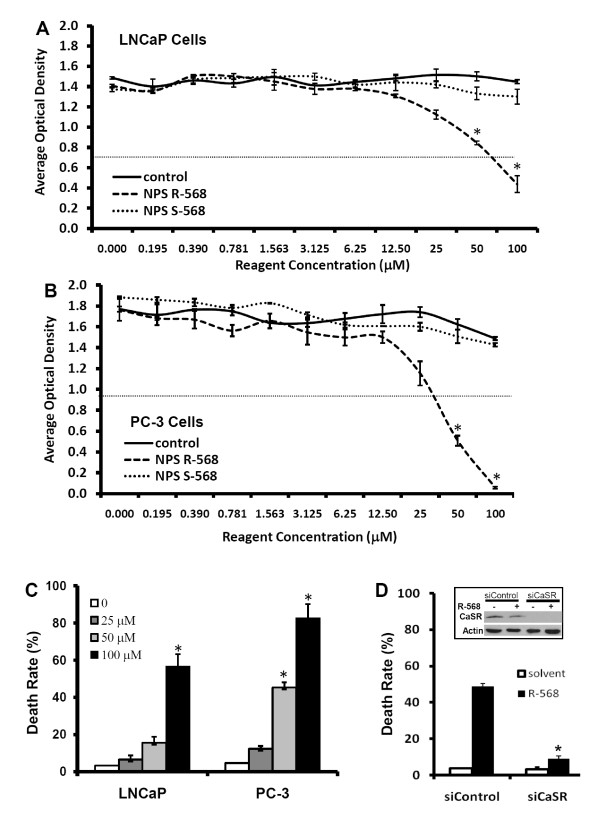
**R-568 reduces cell viability in prostate cancer cells**. **A&B **Cells were seeded in 96-well plates overnight and then treated with R-568 or S-568 at the indicated doses. Control cells received no treatment. After 48 h, viable cells were determined using a MTT assay kit (Sigma, St Louise, MO). The average values of optical densities from each group were presented. Data represents three separate experiments. The red dotted line indicates the IC50 value. **C **Cells were plated in 12-well plates and treated with R-568 at the indicated doses for 48 h. The control cells received no treatment. Cells were harvested at the end of experiment and stained in 0.4% trypan blue solution. The dead (blue) cells were counted and the average of death rate in each well was presented. **D **PC-3 cells were plated in 6-well plates and then transfected with negative control siRNA or CaSR siRNA at 100 μM final concentration in the culture media. Two days later, cells were treated with the solvent or R-568 (50 μM) for 48 hours. Cell death rate was assessed using trypan blue exclusion assay as described earlier. INSERT: Two days after the siRNA transfection, PC-3 cells were treated with or without R-568 for 48 h. Cell lysates were subjected to Western blot for assessing CaSR protein levels. Actin blot served as protein loading control. Data represents three different experiments. The asterisk indicates a significant difference (P < 0.05, Student *t*-test) between R-568 treatment and the control.

The calcimimetic agent R-568 has been shown to activate CaSR and to induce apoptotic cell death in parathyroid cells in addition to reducing PTH secretion [1-3]. In this study, we evaluated R-568-induced effect on cellular survival in two prostate cancer cell lines, androgen receptor-positive LNCaP cells and androgen receptor-negative PC-3 cells. LNCaP cells were derived from lymph node metastasis of prostate cancer, while PC-3 cell line was established from a bone metastasis of human prostate cancer. In a MTT assay, as shown in Fig 1A &1B, the calcimimetic R-568 but not its negative isomer S-568, which does not activate CaSR, significantly reduced cellular viability in both LNCaP and PC-3 cells, of which PC-3 showed a higher sensitivity to R-568 treatment compared to LNCaP cells. In a trypan blue exclusive assay, R-568 treatment exhibited similar cytotoxicity in both LNCaP and PC-3 cell lines in a dose-dependent manner (Fig 1C). However, silencing the CaSR significantly attenuated R-568-induced cell death as compared to the negative siRNA in PC-3 cells (Fig 1D). These data demonstrated for the first time that the calcimimetic agent R-568 is capable of inducing cell death in prostate cancer cells, regardless the status of androgen receptor gene expression, and CaSR activation might play an essential role in R-568-induced cell death.

**Figure 2:**
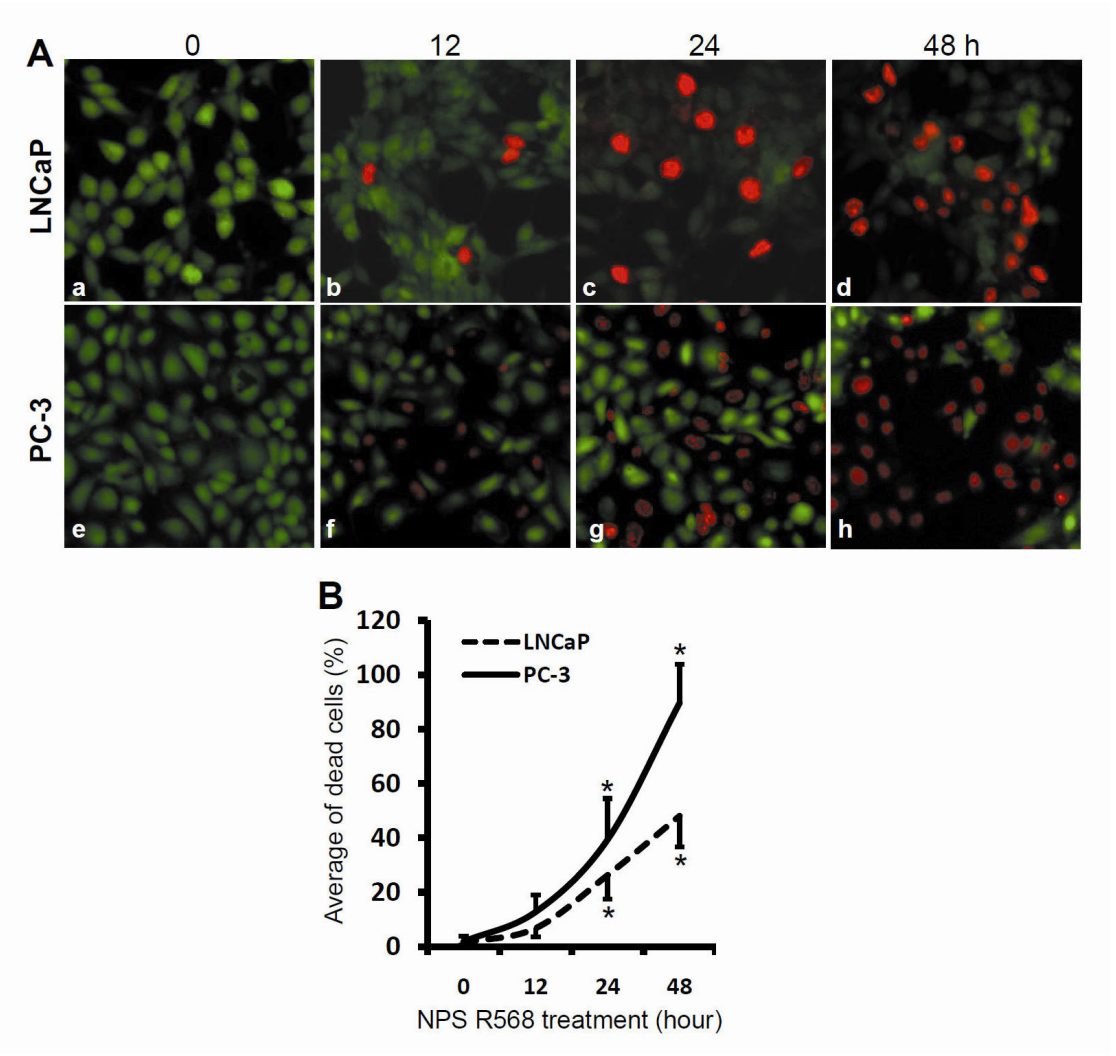
**R-568 induces cell death in prostate cancer cells**. LNCaP and PC-3 cells were plated in 24-well plates overnight, and then treated with R-568 (100 μM) for up to 48 h. At each time points as indicated, the fluorescent dyes (2.0 μM) were added into the culture media and cells were incubated for 15 min before micro-images were taken under a fluorescent microscope (panel A, magnification × 200). Quantitative data for the percentage of dead cells (red-labeled cells) in the total cells (red plus green cells) were summarized in panel B as mean ± SEM from 5 microscopic fields). The asterisk indicates a significant difference (P < 0.01, Student *t*-test) as compared to the value at the 0 hour time point.

To further illustrate the death inducing effect induced by R-568 treatment, we utilized a Live/Dead assay to objectively evaluate cell death. As shown in Fig 2, both cell lines of LNCaP and PC-3 cells showed a time-dependent death response after treatment with R-568 (100 μM). These data confirmed R-568-induced cell death in prostate cancer cells.

### The calcimimetic R-568-induced cell death is an apoptotic event in prostate cancer cells

**Figure 3:**
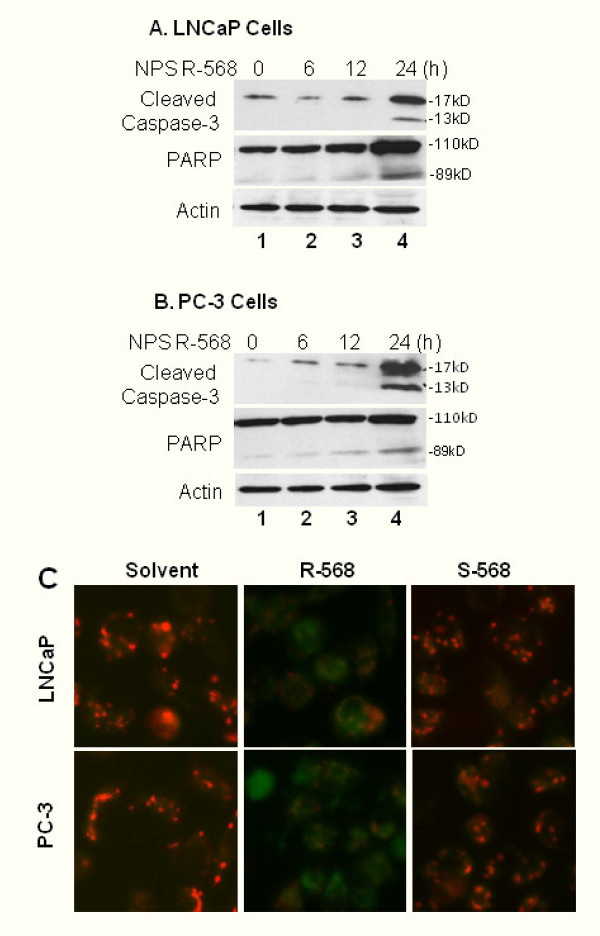
**R-568-induced cell death is an apoptotic response in prostate cancer cells**. **A&B **LNCaP and PC-3 cells were treated with R-568 (50 μM) for different time period as indicated. Equal amounts of cellular proteins were subjected to Western blot assay to assess caspase-3 processing and PARP cleavage. Primary antibodies used are indicated on the left side. Actin blot served as the protein loading control. Data represent two different experiments. **C **LNCaP and PC-3 cells were seeded in 8-well chambered glass slides overnight. Following treatment with R-568 or S-568 at a dose of 50 μM for 24 h, cells were incubated with JC-1 (0.3 μg/ml) for 15 min at 37C. Pictures were taken under a fluorescent microscope. Magnification × 200.

It has been shown that CaSR activation is involved in osteoblast cell apoptosis [4] and R-568 treatment induces apoptotic cell death in rat parathyroid cell [3]. Therefore, we asked if R-568-induced cell death was an apoptotic response in LNCaP and PC-3 cells. We utilized the most commonly used apoptotic markers, caspase-3 processing and PARP cleavage, in our next experiments. As shown in Fig 3 (panel A and panel B), R-568 treatment resulted in a remarkable processing of caspase-3 and a clear pattern of PARP cleavage in both LNCaP and PC-3 cells, indicating that R-568-induced cell death is an apoptotic response.

To further characterize R-568-induced apoptosis, we examined the change of mitochondrial membrane potential using the JC-1 dye, which accumulates in the mitochondria of viable cells as aggregates, which are fluorescent red in color. Conversely, in apoptotic cells, the mitochondrial potential collapses and the JC-1 dye could no longer accumulate in the mitochondria and remains in the cytoplasm in a monomeric form which fluoresces green. As shown in Fig 3C, treatment with R-568 but not S-568 induced a dramatic change of JC-1 color/distribution from red/puncture pattern to green/defused pattern, suggesting that R-568 treatment induced a severe damage to mitochondria, which is consistent with the data shown in Fig 3A and Fig 3B. Taken together, these data strongly suggest that the calcimimetic agent R-568 induced apoptotic cell death *via *a mitochondria-related mechanism.

### The calcimimetic R-568-induced apoptosis is modulated by anti-apoptotic protein Bcl-xL

**Figure 4:**
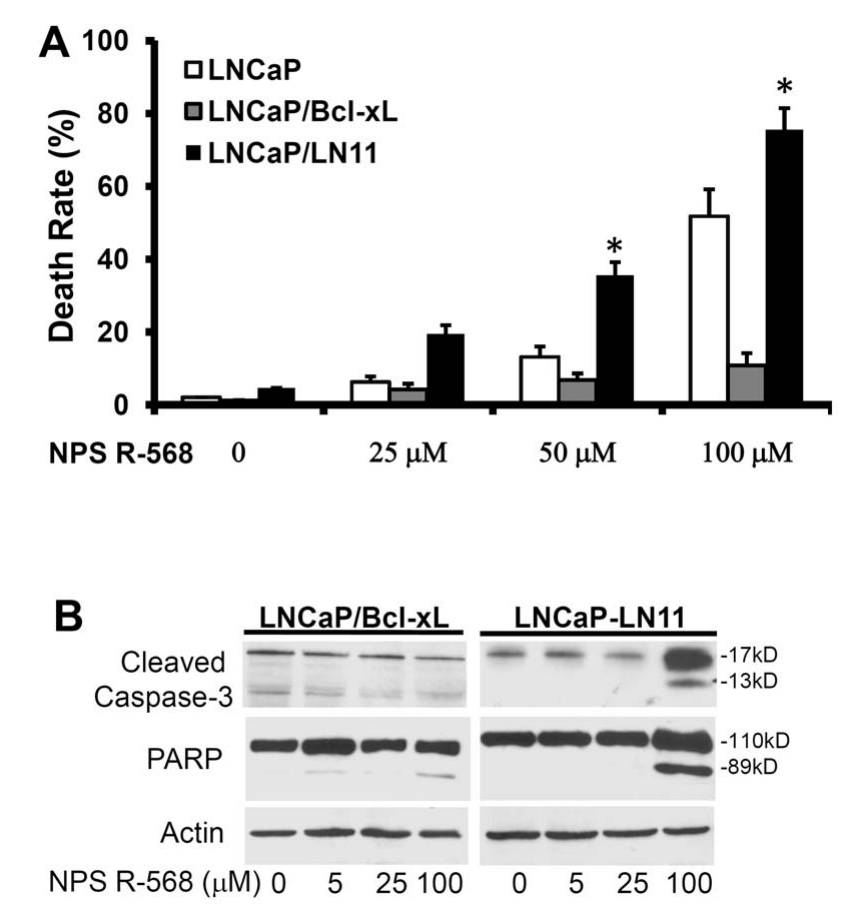
**R-568-induced apoptosis is attenuated by altered Bcl-xL expression in prostate cancer cells**. **A **LNCaP cells and its two sublines, LNCaP/Bclxl and LNCaP/LN11, were seeded in 12-well plates and treated with R-568 at the indicated doses for 48 h. The control cells received no treatment. Cells were harvested at the end of experiment and stained in 0.4% trypan blue solution. The dead (blue) cells were counted and the average of death rate in each well was presented. Data represent three different experiments. The asterisk indicates a significant difference (P < 0.05) between R-568 treatment and the control. **B **LNCaP/Bclxl and LNCaP/LN11 cells were treated with R-568 at indicated doses for 24 h and then harvested for protein extraction. Equal amounts of cellular proteins were subjected to Western blot assay to assess caspase-3 processing and PARP cleavage. Primary antibodies used are indicated on the left side. Actin blot served as the protein loading control. Data represent two different experiments.

Anti-apoptotic protein Bcl-xL is mainly localized on the mitochondrial membrane and plays an important role in maintenance of membrane potential [12]. Recent studies from our group and others showed that Bcl-xL is a major cellular survival factor in castration-resistant prostate cancers [11,13-15]. Therefore, we evaluated if Bcl-xL modulates R-568-induced apoptosis. Two previously confirmed LNCaP sublines, LNCaP/Bclxl (Bcl-xL overexpression) and LNCaP/LN11 (Bcl-xL null) described in our recent publication [11], were used in a trypan blue exclusion assay. Compared to the parental LNCaP cells, enforced Bcl-xL expression abolished R-568-induced cell death in LNCaP/Bclxl cells while loss of Bcl-xL expression significantly increased R-568-induced cell death in LNCaP/LN11 cells [Fig 4A]. Consistently, caspase-3 processing and PARP cleavage were also dramatically attenuated due to altered levels of Bcl-xL expression in response to R-568 treatment [Fig 4B]. These data further confirmed that R-568-induced cytotoxicity is due to mitochondria-related mechanism in prostate cancer cells.

## Discussion

The primary goal of this study was to determine the biological effect of the calcimimetic NPS R-568 on prostate cancer cells. Using two commonly used prostate cancer cell lines, AR-positive LNCaP and AR-negative PC-3, we demonstrated that R-568 reduced cell viability of both cell lines in a dose- and time-dependent manner. R-568-induced cell death is an apoptotic response through a mitochondria-related mechanism and CaSR is essential for R-568-induced cell death. These data provided the preliminary evidence that the calcimimetic R-568 might be useful as adjunctive therapeutic agent for advanced prostate cancers although further pre-clinical testing is desirable.

Currently, limited information is available for calcimimetic NPS R-568-induced apoptosis in mammalian cells. In this study, we showed that R-568 treatment disrupted mitochondrial membrane potential and that modulation of the anti-apoptotic protein Bcl-xL expression attenuated R-568-induced caspase-3 activation and cell death, suggesting that an intrinsic apoptosis pathway is triggered by R-568 treatment. In both LNCaP and PC-3 cells, R-568-induced cell death was found in a range of concentrations that are similar to the doses used in a recent report to induce apoptosis in isolated rat parathyroid cells [3]. The calcimimetic agents have been reported to increase intracellular calcium concentration in a dose-dependent manner [16], and calcium accumulation in mitochondria has been considered as a major apoptotic mechanism [reviewed in ref. [17]]. Thus, it is plausible that R-568 increased cytosolic calcium, leading to calcium accumulation and mitochondrial stress, eventually resulting in apoptotic cell death. Further investigation in this aspect is underway by our group.

CaSR signaling has been studied in multiple cancers and different effects were reported depending on the cell types and agonists used [reviewed in ref. [18]]. For example, in parathyroid adenoma and colon cancers, loss of CaSR expression was reported, leading to uncontrolled growth due to elevated calcium level. In prostate cancers, calcium-mediated CaSR activation was reported to prevent apoptosis [19], and to stimulate cell proliferation [20], and to increase production of PTH-related protein (PTHrP), a causal factor in bone metastasis [9,10]. On the other hand, CaSR-mediated apoptosis was also reported in osteoblast and human embryonic kidney cells [4,21], especially the calcimimetic R-568-induced apoptotic cell death in hyperplastic parathyroid cells [3]. Consistently, in this study, we provided the first evidence that R-568 but not its negative isomer S-568 induces apoptotic cell death in human prostate cancer cells, and that R-568-induced cell death is *via *a CaSR-dependent pathway.

In conclusion, we demonstrated that the calcimimetic R-568 induces apoptotic cell death in prostate cancer cells. R-568-induced apoptotic cell death is *via *a mitochondria-related pathway. The usefulness of the calcimimetic agent in managing prostate cancer patients needs further testing in pre-clinical and clinical study.

## Abbreviations

AR: androgen receptor; CaSR: calcium sensing receptor; FBS: fetal bovine serum; MTT: [3-[4,5-dimethylthiazol-2-yl]-2,5-diphenyltetrazolium bromide]; PARP: poly [ADP-ribose] polymerase; PBS: phosphate-buffered saline; PTH: parathyroid hormone; PTHrP: PTH-related protein; SEM: standard error of mean; SHPT: secondary hyperparathyroidism; TBS-T: Tris-buffered solution plus Tween 20.

## Competing interests

The authors declare that they have no competing interests.

## Authors' contributions

HL, BL and MZ designed the experiments, HL, GR participated in most of the experiments, ZL and XZ carried out the siRNA experiments, HZ and GC conducted the JC-1 experiments, HL and MZ drafted the manuscript. BL was involved in design of the study and performed the statistical analysis and helped to finalize the manuscript. All authors read and approved the final manuscript.
